# Immunological response to quadrivalent HPV vaccine in treatment of recurrent respiratory papillomatosis

**DOI:** 10.1007/s00405-016-4085-3

**Published:** 2016-05-17

**Authors:** Robin E. A. Tjon Pian Gi, Michel R. M. San Giorgi, Michael Pawlita, Angelika Michel, Bettien M. van Hemel, Ed M. D. Schuuring, Edwin R. van den Heuvel, Bernard F. A. M. van der Laan, Frederik G. Dikkers

**Affiliations:** 1 Department of Otorhinolaryngology/Head and Neck Surgery, University of Groningen, University Medical Center Groningen, P.O. Box 30.001, 9700 RB Groningen, The Netherlands; 2 Division of Molecular Diagnostics of Oncogenic Infections, German Cancer Research Center, Heidelberg, Germany; 3 Department of Pathology, University of Groningen, University Medical Center Groningen, Groningen, The Netherlands; 4 Department of Mathematics and Computer Science, Eindhoven University of Technology, Eindhoven, The Netherlands; 5 Graduate School of Medical Sciences (Cancer Research Center Groningen), University of Groningen, Groningen, The Netherlands

**Keywords:** Recurrent respiratory papillomatosis, Vaccine, Therapeutic drug, Immunology

## Abstract

Aim of this study was to explore influence of the quadrivalent HPV vaccine (Gardasil^®^) on the immune status of recurrent respiratory papillomatosis (RRP) patients. In retrospective observational study, six RRP patients who received the quadrivalent HPV vaccine and whose HPV seroreactivity was measured were included. Multiplex HPV Serology was used to determine HPV-specific antibodies pre- and post-vaccination. Surgical interventions and patient records were analyzed. Five HPV6 and 1 HPV11 infected patient were included. Mean antibody reactivity against the associated HPV type rose from 1125 median fluorescence intensity (MFI) pre-vaccination to 4690 MFI post-vaccination (*p* < 0.001). Median post-vaccination follow-up was 4 years. Poisson regression analysis showed that the quadrivalent HPV vaccine decreased the incidence rate of surgeries. The immune system of RRP patients is able to increase antibody reactivity against the associated HPV type. A double blind randomized controlled trial is needed to determine whether this immunological increase can cause decrease in number of surgeries.

## Introduction

Infection with a subset of human papillomaviruses (HPV) can cause anogenital cancer, oropharyngeal cancer, condylomata acuminata and recurrent respiratory papillomatosis (RRP) [1, 2]. Since 2006 many national vaccination programs have started with the bivalent HPV vaccine (Cervarix^®^, GlaxoSmithKline Biologicals s.a., Rixensart, Belgium) or the quadrivalent HPV vaccine (Gardasil^®^, Merck & co, Whitehouse Station, USA) targeting high-risk oncogenic HPV types 16 and 18. Papillomavirus vaccines are generally safe and highly effective [3]. The quadrivalent HPV vaccine is a subunit vaccine composed of the major capsid protein L1 primarily in the form of virus-like particles (VLPs) of low-risk HPV6 and 11 and high-risk HPV16 and 18. HPV6 and 11 cause 90 % of genital warts [4]. It is expected that preventive global use of this HPV vaccine against cervical cancer will decrease the incidence of HPV6 and HPV11 related disease worldwide [5].

Recurrent respiratory papillomatosis (RRP) is a wart-like disease characterized by its unpredictable clinical course. It is associated with HPV6 and 11 for 80–100 % of cases [6–10]. Therapy focuses on repeated surgical removal of exophytic lesions. Some patients may need over a 100 surgical interventions to keep the airway open and the voice sufficient [6].

Antibody response after vaccination with the quadrivalent HPV vaccine is higher than after natural infection in patients with high-risk HPV [3, 11]. Little is known about the antibody response for low-risk HPV. Increased seroreactivity after vaccination in RRP patients was only addressed in two case reports [12, 13]. After vaccination with the quadrivalent HPV vaccine, HPV seropositive women were protected against cervical and anogenital diseases from the corresponding HPV type [3]. Therefore, vaccination of RRP patients could be a potential treatment against HPV re-infection or auto-inoculation. In this independent exploratory study we investigated whether vaccination with the quadrivalent HPV vaccine results in increase of antibodies against the associated viruses.

## Materials and methods

### Ethical considerations

Patients included in this retrospective cohort study were clinically treated off-label with the quadrivalent HPV vaccine; there was no scientific intent. Due to great international interest in the use of this therapy, it was decided to publish these valuable data. Written approval of all patients was received.

Institutional Review Board approval for retrospective cohort research is not needed in The Netherlands. All patients approved use of information from their patient files, laboratory results and biopsy material, by signing a consent form.

Biopsy and resection material of the included patients were available in archives of our Department of Pathology. This study was performed according to the Code of Conduct for Proper Secondary Use of Human Tissue in The Netherlands, as well as to the applicable institutional and national guidelines [14].

### Patients

Patients’ charts and surgical reports of all RRP patients treated at the Department of Otorhinolaryngology/Head and Neck Surgery of the tertiary referral hospital University Medical Center Groningen, University of Groningen, The Netherlands were retrospectively analyzed. Inclusion criteria for this study were: (1) histological confirmation of RRP by an experienced head and neck pathologist, (2) the patient received the quadrivalent HPV vaccine with therapeutic intent, (3) HPV seroreactivity known pre- and post-vaccination.

Patient charts were reviewed on date of birth, gender, date of diagnosis, risk factors (gastroesophageal reflux disease (GERD) and asthma), follow-up, number of surgeries, complications of administration of the quadrivalent HPV vaccine and complications associated with RRP (carcinoma, tracheotomy). Patients with an age of onset younger than 18 years of age have juvenile onset RRP (JoRRP). Patients older than 18 years at onset of disease have adult onset RRP (AoRRP).

### Vaccination

The quadrivalent HPV vaccine was clinically administered to RRP patients between March 2011 and January 2013. Vaccination was injected intramuscularly by normal dosage of VLP6 20 μg, VLP11 40 μg, VLP16 40 μg, and VLP18 20 μg per injection (0.5 ml). Injections were given following the same schedule as in preventive vaccination: at 0, 2 and 6 months [15]. The time after the first administration was considered as ‘post-vaccination’. The second and third vaccinations were administered for durability of the effect [15].

### Time frame

A blood sample was taken immediately before the first injection of the quadrivalent HPV vaccine (pre-vaccination seroreactivity). A second blood sample was taken immediately before the third vaccination (representing post-vaccination seroreactivity).

### Patient material

#### HPV type specific polymerase chain reaction (PCR)

For each patient a stored paraffin block from the first formalin-fixed biopsy was selected, in which papilloma was histopathologically confirmed. To confirm presence of papilloma an experienced pathologist revised all biopsies. When quality or quantity of the first biopsy was not sufficient for PCR, the next sufficient biopsy was used. HPV typing was performed using the HPV consensus primer set GP5+/6+ with subsequent nucleotide sequence analysis. Details of this technique have been described before [6].

#### Antibody seroreactivity

Seroreactivity to the HPV major capsid L1 protein for both HPV6 and HPV11 was measured to monitor antibody response against the quadrivalent HPV vaccine. Blood samples were analyzed by the multiplex human papillomavirus serology, based on in situ-purified glutathione S-transferase proteins, as described by Waterboer et al. [16, 17]. Briefly, full-length L1 proteins were bacterially expressed as fusion proteins with N terminal glutathione-S-transferase (GST) and a C terminal tagging peptide (tag) and were affinity-purified in situ from cleared bacterial lysates through binding to glutathione casein-coated fluorescence-labeled polystyrene beads. A fusion protein consisting of GST and tag (GST-tag) without intervening viral antigen served for background determination. Each fusion protein was bound to a spectrally distinct bead set. Fusion protein-loaded bead sets were mixed. Sera were pre-incubated at 1:50 dilution in PBS containing 1 mg/mL casein, 2 mg/mL lysate from bacteria expressing GST-tag alone to block antibodies directed against residual bacterial proteins and GST-tag, 0.5 % polyvinylalcohol (PVA, Sigma-Aldrich Chemie Gmbh Munich, Germany), 0.8 % polyvinylpyrrolidone (PVP, Sigma-Aldrich Chemie Gmbh Munich, Germany) and 2.5 % Superchemiblock (Millipore, Billerica, MA, USA) to suppress unspecific binding of antibodies to the beads themselves [17]. Serum dilutions were incubated with the same volume of mixed bead sets, resulting in a final serum dilution of 1:100. Bound antibodies were detected with biotinylated goat-antihuman IgG (H + L) secondary antibody and streptavidin-*R*-phycoerythrin. A Luminex analyser (xMAP, Luminex Corp., Austin, TX, USA) was used to identify the internal color of the individual beads and to quantify their reporter fluorescence (expressed as median fluorescence intensity (MFI) of at least 100 beads per set per serum). Antibody reactivity, i.e. the amount of antigen-specific antibody bound per bead is expressed as net MFI values calculated as difference of MFI with HPV-protein minus MFI with GST-tag.

### Statistical analysis

Analyses were performed using PASW statistics version 20.0 (SPSS Inc., Chicago, IL, USA) and SAS version 9.3 (SAS Institute, Inc., Cary, NC, USA).

Categorical variables are presented as number (percentage). Normally distributed variables are presented as mean ± standard deviation. Data presented as [*x*; *x*] represents 95 % confidence interval. *p* value of <0.05 was considered statistically significant.

A paired *t* test was used to analyze the difference between pre- and post MFI. Descriptive statistics were provided for the rate of surgical interventions (number of surgical interventions divided by time interval) before and after vaccinations. A Spearman correlation coefficient between the two rates was calculated. Poisson regression analysis (with a random intercept for subjects) was applied to investigate a possible effect of vaccine on the mean number of surgical interventions corrected for type of papilloma virus (HPV6 and HPV11) and age at onset. Subject’s variable log time period was included in the regression analysis as offset parameter to adjust for different time intervals for subjects.

Since the analysis is only preliminary and exploratory, a sample size for a parallel group randomized clinical trial was calculated on the basis of an effect size that vaccination reduces the mean number of surgical interventions with 50 %. Formula four of Signorini et al. with a Bernoulli covariate was used [18].

## Results

Nine RRP patients of the University Medical Center Groningen received the quadrivalent HPV vaccine. For six of them seroreactivity pre- and post-vaccination were known; these six patients were included in this exploratory study. Patients were diagnosed with RRP between 1981 and 2011, followed until August 1, 2015. Characteristics per patient are presented in Table 1. All included patients were male. The mean age of onset was 16 years (SD 16). Three patients (50 %) had JoRRP, three patients (50 %) had AoRRP. None of the patients had asthma or GERD. Five patients were infected with HPV6 and one patient was infected with HPV11.

**Table 1:** Characteristics per patient, pre- and post-vaccination

Patient ID	Gender (M/F)	HPV type	Age of onset (years)—age at first vaccination (years)	JoRRP/AoRRP	Asthma	GERD	Smoker	Tracheostomy	Cidofovir	Pre-vaccination follow-up (years)	Pre-vaccination surgeries (*n*)	Pre-vaccination antibody reactivity (MFI)	Post-vaccination follow-up (years)	Post-vaccination surgeries (*n)*	Post-vaccination antibody reactivity (MFI)
#1	M	11	2–33	JoRRP	−	−	−	+	+	30	79	171	3	7	4841
#2	M	6	39–46	AoRRP	−	−	−	−	+	7	9	1887	4	2	5516
#3	M	6	4–9	JoRRP	−	−	−	−	−	5	4	2422	3	5	3621
#4	M	6	29–31	AoRRP	−	−	+	−	+	2	11	925	4	1	5419
#5	M	6	21–23	AoRRP	−	−	−	−	+	2	11	1048	4	2	4549
#6	M	6	2–4	JoRRP	−	−	−	−	+	1	7	297	4	5	4199

*M* male, *F* female, *JoRRP* juvenile onset recurrent respiratory papillomatosis, *AoRRP* adult onset recurrent respiratory papillomatosis, *GERD* gastroesophageal reflux disease, *MFI* mean fluorescence intensity, *Cidofovir* cidofovir in history

**Fig. 1:**
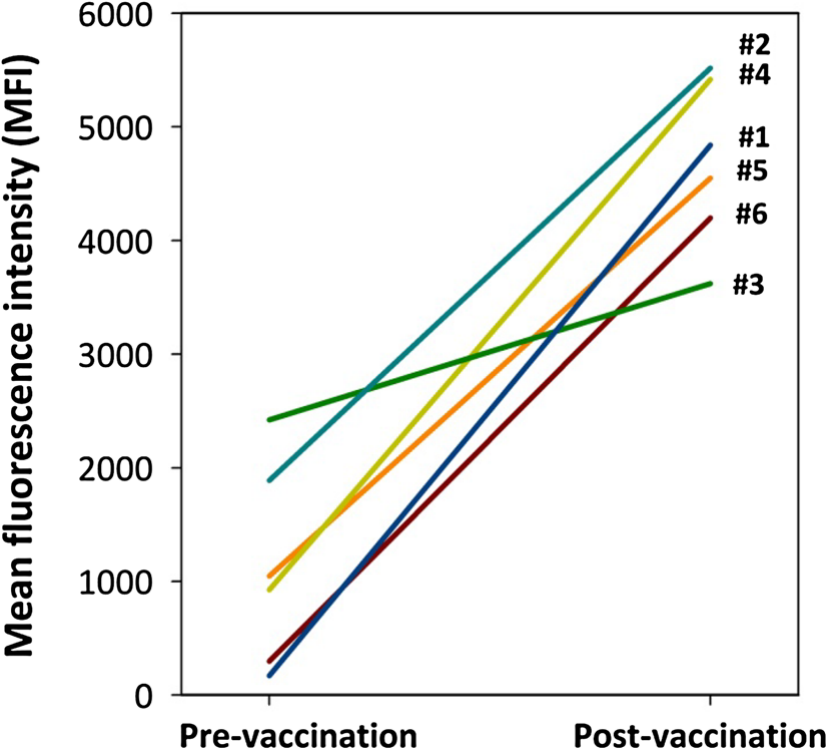
Antibody reactivity per patient against the associated HPV type pre- and post-vaccination (*#1* HPV11 patient, *#2*–*#6* HPV6 patients)

The mean pre-vaccination antibody reactivity was 1125 MFI (SD 884). The mean post-vaccination antibody reactivity was 4690 MFI (SD 727). All individual antibody reactivities increased after vaccination, with a median rise of 3766 MFI (range 1199–4670). The mean MFI per patient rose significantly after vaccination (*p* < 0.001). The change of pre- and post-vaccination antibody reactivity of the associated viruses are represented in Fig. 1.

**Fig. 2:**
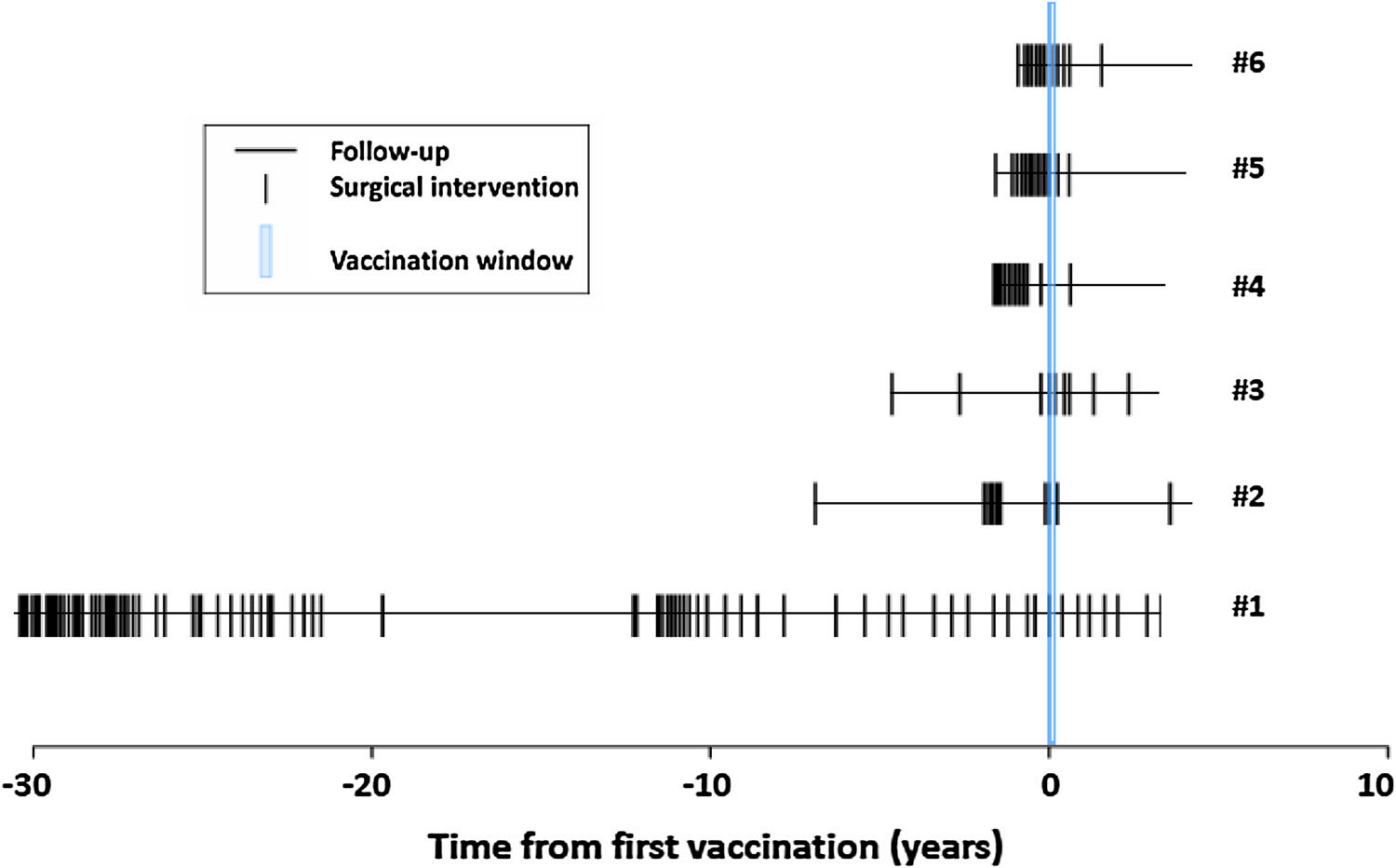
Follow-up with all surgical interventions by age of the patient (*n* = 6), pre- and post-vaccination. Vaccinations were administered during the *blue-marked* period. *#1* HPV11 patient, *#2*–*#6* HPV6 patients

None of the patients experienced side effects or complications of the vaccination. The surgical course over time is presented in Fig. 2. The median pre-vaccination disease history was 3 years (range 1–30). The median post-vaccination follow-up was 4 years (range 3–4). The interval between surgeries ranged from 1 week to 7 years (Fig. 2). The average rates of surgical interventions for a period of a year were 4.34 [1.11; 7.57] and 0.99 [0.25; 1.73] before and after vaccination, respectively. Spearman correlation coefficient between the rates before and after was estimated at −0.20 (*p* = 0.704).

Poisson regression analysis corrected for age at onset and type of HPV demonstrated a clinical effect of vaccination. The effect size was estimated at −1.20 [−1.90; −0.50]. This meant that the mean number of surgical interventions in a specific time frame after vaccination decreased with approximately a factor of 3.3 (=exp^1.20^).

Based on the results of a simpler Poisson regression analysis (using only the vaccination variable and overdispersion), the sample size for detecting reduction in the mean number of surgical interventions after vaccination with a factor of 2 was calculated. If a theoretical trial period would be 1 year, the total number of patients in each group should be 57. If a trial would be extended to 1.5 years, the number of patients in each group should be 38, while for a trial of 2 years the number of patients should be 29 in each group.

## Discussion

Many therapies have been tried to diminish disease burden of RRP. Nonetheless there is still no curative therapy for RRP patients. The primary goal of this exploratory study was to monitor effectiveness of the quadrivalent HPV vaccine during treatment of RRP as determined by increased seroreactivity. This is the first study that shows that vaccination of a group of RRP patients with the quadrivalent HPV vaccine results in increased seroreactivity against associated viruses.

Five of six patients were infected with low-risk HPV6, one was infected with low-risk HPV11. The ratio between HPV6 and HPV11 differs per cohort [19], probably because of geographical spread of both viruses [20]. This research consisted of both JoRRP and AoRRP patients. The immunological response is therefore representable for both groups. A difference in immune response is not expected. RRP patients with a pre-vaccination history of 1–30 years were included.

The presented data show that RRP patients with HPV6 and HPV11 have low levels of seroreactivity against these viruses despite many years of disease. After administration of the quadrivalent HPV vaccine HPV seroreactivity against the causal HPV type rose in every patient. The vaccination induced higher seroreactivity than the natural infection in the same patients. Theoretically this induced increase in seroreactivity might influence the clinical course of RRP by intensifying immune response and preventing re-infection.

This study is the first study to measure seroreactivity in a group of RRP patients. Results could be biased due to the small sample size and short follow-up. An effect of other adjuvant therapy on immunological response was not expected as patients did not receive any adjuvant therapy 1 month before, neither during or after vaccination. More research is needed to analyze the duration of the immunological response.

Chirila et al. concluded that the quadrivalent HPV vaccine was effective to diminish the recurrence rate of RRP in 85 % of patients, although that study was retrospective and lacked a control group [21]. Furthermore the natural decreasing surgical rate of RRP was not taken into account [6, 22]. It is unknown if there is a immune response after vaccination which explains a clinical response, therefore this study was conducted. The clinical response described in this article was only used for a power analysis for a future randomized controlled trial (RCT), as the sample size was too small to analyze the clinical course and to correct for the natural clinical course and other therapies (e.g. cidofovir). A RCT is needed to draw conclusions on the real clinical effect of the quadrivalent HPV vaccine. The proposed sample size for a trial with a follow-up of 2 years should be 29 patients per group.

## Conclusion

RRP patients increase seroreactivity against the quadrivalent HPV vaccine, regardless of their age, age of onset, HPV type and severity of disease. Antibody reactivities to the associated viruses of all patients rose significantly. A double-blinded randomized controlled trial is needed to evaluate the effect of this vaccination on the clinical course. The quadrivalent HPV vaccine could be of future help in the treatment of RRP, as this research showed that vaccination causes a robust immunological response.
